# Predicting Psychotic Relapse in Schizophrenia With Mobile Sensor Data: Routine Cluster Analysis

**DOI:** 10.2196/31006

**Published:** 2022-04-11

**Authors:** Joanne Zhou, Bishal Lamichhane, Dror Ben-Zeev, Andrew Campbell, Akane Sano

**Affiliations:** 1 Department of Statistics Rice University Houston, TX United States; 2 Department of Electrical and Computer Engineering Rice University Houston, TX United States; 3 Behavioral Research in Technology and Engineering Center Psychiatry and Behavioral Sciences University of Washington Seattle, WA United States; 4 Department of Computer Science Dartmouth College Hanover, NH United States

**Keywords:** schizophrenia, psychotic relapse, machine learning, clustering, mobile phone, routine, Gaussian mixture models, partition around medoids, dynamic time warping, balanced random forest

## Abstract

**Background:**

Behavioral representations obtained from mobile sensing data can be helpful for the prediction of an oncoming psychotic relapse in patients with schizophrenia and the delivery of timely interventions to mitigate such relapse.

**Objective:**

In this study, we aim to develop clustering models to obtain behavioral representations from continuous multimodal mobile sensing data for relapse prediction tasks. The identified clusters can represent different routine behavioral trends related to daily living of patients and atypical behavioral trends associated with impending relapse.

**Methods:**

We used the mobile sensing data obtained from the CrossCheck project for our analysis. Continuous data from six different mobile sensing-based modalities (ambient light, sound, conversation, acceleration, etc) obtained from 63 patients with schizophrenia, each monitored for up to a year, were used for the clustering models and relapse prediction evaluation. Two clustering models, Gaussian mixture model (GMM) and partition around medoids (PAM), were used to obtain behavioral representations from the mobile sensing data. These models have different notions of similarity between behaviors as represented by the mobile sensing data, and thus, provide different behavioral characterizations. The features obtained from the clustering models were used to train and evaluate a personalized relapse prediction model using balanced random forest. The personalization was performed by identifying optimal features for a given patient based on a personalization subset consisting of other patients of similar age.

**Results:**

The clusters identified using the GMM and PAM models were found to represent different behavioral patterns (such as clusters representing sedentary days, active days but with low communication, etc). Although GMM-based models better characterized routine behaviors by discovering dense clusters with low cluster spread, some other identified clusters had a larger cluster spread, likely indicating heterogeneous behavioral characterizations. On the other hand, PAM model-based clusters had lower variability of cluster spread, indicating more homogeneous behavioral characterization in the obtained clusters. Significant changes near the relapse periods were observed in the obtained behavioral representation features from the clustering models. The clustering model-based features, together with other features characterizing the mobile sensing data, resulted in an F2 score of 0.23 for the relapse prediction task in a leave-one-patient-out evaluation setting. The obtained F2 score was significantly higher than that of a random classification baseline with an average F2 score of 0.042.

**Conclusions:**

Mobile sensing can capture behavioral trends using different sensing modalities. Clustering of the daily mobile sensing data may help discover routine and atypical behavioral trends. In this study, we used GMM-based and PAM-based cluster models to obtain behavioral trends in patients with schizophrenia. The features derived from the cluster models were found to be predictive for detecting an oncoming psychotic relapse. Such relapse prediction models can be helpful in enabling timely interventions.

## Introduction

### Background

Schizophrenia is the most common psychotic disorder, affecting up to 20 million people worldwide [1] and accounting for more than 13.4 million years of life lived with a disability [2]. It can be caused by a combination of genetic, environmental, and psychosocial factors. Patients with schizophrenia experience a range of positive symptoms (hallucinations, delusions, etc), negative symptoms (anhedonia, social withdrawal, etc), and cognitive dysfunctions (lack of attention, working memory, executive function, etc) [3,4]. The disorder is highly disabling and often has consequences such as impairment of education, employment, and social contact [4]. Adults with schizophrenia also have an increased risk of premature mortality than the general population [5]. Therefore, proper treatment and management of schizophrenia are important to limit the negative impact of the disorder on the individual’s life.

Schizophrenia is usually treated with a combination of antipsychotic medications and psychosocial treatments. However, patients undergoing treatment can still experience psychotic or symptomatic relapse, an acute exacerbation of schizophrenia symptoms [6]. A previous study found that the cumulative first and second relapse rates were 81.9% and 78%, respectively, within 5 years of recovery from the first episode of schizophrenia and schizoaffective disorder [7]. The risk of relapse is found to be significantly higher after treatment reduction or discontinuation [6]. Relapse poses severe health risks for the individuals and can jeopardize their treatment progression and daily functioning. Each relapse episode is associated with a risk of self-harm and harm to others [8].

To monitor a patient’s health status and recovery, routine clinic visits for continual assessment are recommended. Clinical interviews and questionnaire tools were used during the visit to assess current health symptoms and provide timely intervention to prevent relapses [9]. However, relapses may occur between the visits, during which a patient’s health status is not assessed. In addition, patients may have limited insight during a psychotic relapse and may struggle to report it to the treatment team or a significant other. Therefore, improving treatment adherence and preventing relapses have become a focus of schizophrenia management. Toward the effort of relapse prevention, there has been significant interest in mobile sensing-based behavioral monitoring models for automatic relapse risk prediction.

### Previous Studies

Smartphone apps and wearable devices have been used in several previous studies to collect passive sensing data and track daily behaviors, which can then be used to model the relationship between behaviors and mental well-being. For example, in the StudentLife study, an Android sensing app collected passive sensing data from 48 college students, and the inferred behavioral features from the collected data were found to be correlated with academic performance and self-reported mental health conditions [10]. In a study on depression severity, the mobile sensing-based features, such as daily behavioral rhythms, variance of patient’s location, and phone use, were found to be related to depressive symptom severity [11]. The use of mobile sensing to collect long-term monitoring data has also been demonstrated to be feasible and acceptable for patients with schizophrenia disorders [12-15]. Surveys have found that people with schizophrenia commonly access digital devices for communication and support related to the disorder, which again shows the applicability of using mobile sensing as a platform to monitor schizophrenia symptoms [16].

Mobile sensing data have been used to model behaviors and predict psychotic relapses in patients with schizophrenia. If an oncoming relapse can be detected with high accuracy, timely medical interventions can be provided to mitigate the associated risks. Researchers have found anomalies in daily behavior assessed from mobile sensing before relapses and developed relapse prediction models with promising accuracy [17-19]. In a pilot study, the Beiwe app collected mobile sensing data from 15 patients with schizophrenia for 3 months, during which 5 patients experienced relapses [17]. The researchers found that the rate of anomalies in mobility and social behavior increased significantly closer to relapses. In the CrossCheck project, a mobile sensing app was developed to collect self-reporting ecological momentary assessment and continuous passive sensing data from 75 outpatients with schizophrenia [20]. On the basis of this data set, Wang et al [18] compared different machine learning models for relapse prediction, with several feature extraction windows, and identified the best classifier and prediction settings for detecting an oncoming relapse. The best performance was obtained using a support vector machine (with radial basis function kernel) model and a feature extraction window of 30 days, leading to an F1 score of 0.27 on the relapse prediction task. Similarly, the Adler et al [21] used an anomaly detection framework based on an encoder-decoder reconstruction loss to predict psychotic relapse in schizophrenia.

Concerning current mental health status, the extent to which an individual adheres to work, sleep, social, or mobility routine (ie, a regular behavioral pattern) largely impacts their mental well-being and symptom severity of mental disorders [11,22,23]. Behavioral stability features that measure the adherence to routines have been proposed as relapse predictors in some of the previous studies. Features computed in our previous study measured behavioral stability by calculating the temporal evolution of daily templates of features derived from mobile sensing data (daily templates are time series obtained with representative feature values at regular time intervals in a given day; eg, time series of hourly feature values) [19]. Tseng et al [24] also showed the effectiveness of using behavioral rhythm-based features to predict different symptom severities. Stability features such as deviation of daily templates were found to be significant predictors of schizophrenia symptoms, such as depression. He-Yueya et al [25] also proposed a stability metric for behaviors with a fine temporal resolution by calculating the distance between 2 cumulative sum functions describing behaviors in a certain minute of the day. The computed stability index had similar predictive power as the state-of-the-art behavioral features (mean and SD of each behavior) in the study by Wang et al [26], while being complementary. In all the previous studies using behavioral stability to model relapse prediction, the measured stability was limited to the behaviors observed within a short feature extraction window (eg, few weeks only). An individual’s routine behaviors were not fully represented owing to the short time window considerations. A summary of behavioral patterns can rather be obtained when large time windows are considered.

In this study, instead of measuring behavioral patterns using the variance of day-to-day behaviors, we identify the overall cluster of behaviors for an individual using multimodal mobile phone data and unsupervised machine learning and derive features based on the distance of behaviors observed in a day compared with the individual’s most representative routines. The identified behavioral clusters for an individual could represent their weekday routine, weekend routine, low-phone-use routine (no sensor reading), and so on. The identified clusters provide a representation of the long-term behavioral trends across the patients, which are not directly captured by short-term behavioral rhythm features, as used in previous studies. Furthermore, clusters obtained from the mobile sensing data represent quantized behaviors, and features derived from these clusters are robust to the insignificant variations in behavior compared with the short-term behavioral rhythm change features. Typical behavioral routines for individuals can be found via the clustering analysis of their daily behaviors. Previously, clustering has been applied for identifying mobility patterns using GPS sensing data and evaluating anomalies accordingly [21,26]. However, to the best of our knowledge, clustering analysis has not been performed for characterizing the overall behavioral patterns of patients with schizophrenia, using multimodal mobile sensing data toward relapse prediction tasks.

### Goal of This Study

In this study, we aim to (1) develop a method to characterize patients’ daily behaviors using multimodal smartphone sensor data, (2) understand the relationship between behavioral patterns and psychotic relapse events in schizophrenia, and (3) evaluate the predictive power of the identified behavioral pattern-based features for relapse prediction. We propose multivariate time series clustering of daily templates obtained from mobile sensing data to obtain behavioral patterns. Then, the features derived from clustering are used in the relapse prediction task. The paper is organized as follows. In the *Methods* section, we describe the method used to cluster multidimensional daily templates from mobile sensing data, model selection approach for clustering, and feature extraction and relapse prediction modeling. In the *Results* section, we present the results obtained from the clustering models, association of the obtained clustering-based behavioral features with relapses, and evaluation of the developed relapse prediction model. The obtained results are discussed and future directions are outlined in the *Discussion* section.

## Methods

### Ethics Approval

This study was approved by the ethical review committee of Dartmouth College (#24356) and the institutional review board of North Shore-Long Island Jewish Health System (#14-100B) [20].

### Data Preparation

The data used in this study were obtained from the CrossCheck project (clinical trial registration: ClinicalTrials.gov, NCT01952041 [27]), which was conducted at the Zucker Hillside Hospital in New York City, New York [20,24,26,28,29]. Informed consents were obtained from the participants. The inclusion criteria for the participants are described in the study by Ben-Zeev et al [20]. The CrossCheck app collected mobile sensing data from 75 outpatients with schizophrenia with a data collection period of >12 months per patient. Of the 75 patients, 63 (84%) patients completed the data collection (n=27, 43% men and n=36, 57% women; average age 37.2, SD 13.7 years; range 18-65 years), and a total of 27 relapse events occurred in 32% (20/63) of the patients during the monitoring period. Some patients had multiple incidences of relapses, but as the monitoring period was long, each of the incidences was treated as a unique event if separated by a month. A relapse incident was defined as one that has occurred under one or more of the following seven criteria: psychiatric hospitalization, increased frequency or intensity of services, increased medications or dosages or >25% changes in Brief Psychiatric Rating Scale scores, suicidal ideation, homicidal ideation, self-injury, and violent behavior resulting in harm to self or others [18]. A total of 6 mobile sensing modalities including physical activity, sociability, and ambient environmental readings were obtained using the app. Different features were extracted from these mobile sensing modalities, as presented by Tseng et al [24]. From these features, a total of 21 passive sensing features were selected for our proposed clustering-based behavioral characterization: acceleration, distance traveled, sleep duration, ambient sound, ambient light, conversation duration, phone unlock duration, and different types of call log, SMS text message log, and app use. All the features were transformed to an hourly resolution by averaging the observations within 1 hour. For features that were obtained with lower resolution (eg, every few hours), for example, distance traveled from morning to noon, the feature values were split to each hour spanned by the time represented by these feature values. With hourly resolution for each of the 21 features considered and these hourly feature values considered as separate feature space, the resulting data set had a dimension of 504 (21×24). Observational data for a total of 18,436 days were collected for all the patients. Per-patient feature normalization (min-max normalization between 0 to 1) was performed to adjust for differences between patients. From the normalized data set, principal component analysis on the full data set (with data from all the patients) was performed for dimensionality reduction. The first 200 principal components were retained, which explained 96.9% of the total variance.

### Clustering Models

We evaluated two different clustering methods: Gaussian mixture model (GMM) and partition around medoids (PAM), to cluster the features from the mobile sensing data and obtain behavioral representations. The 2 clustering models differ in how the similarity between different points are assessed, representing different ways in which behaviors across days can be compared with each other, and therefore, produce different cluster outputs.

#### GMM Clustering

##### Model Introduction

The GMM is a probabilistic model that assumes data are generated from a finite set of Gaussian distributions. Gaussian mixture probability density is the weighted sum of k component Gaussian densities [30]. The GMM can address correlation between attributes by selecting the optimal covariance matrix for each cluster and has been used in previous behavioral clustering problems [31]. Moreover, it can derive the probability of each sample in its assigned Gaussian distribution. In this study, we used the GMM implementation from the scikit-learn package in Python to obtain a clustering model for the mobile sensing data [32]. The parameters of the GMM were obtained using the expectation-maximization algorithm [33]. We selected the number of clusters and covariance matrix type based on Akaike information criterion (AIC) score and Bayesian information criterion (BIC) score of all the candidate models (see more details in Multimedia Appendix 1 [34,35]).

##### Model Output

Three output variables for each of the data points (observations), offering GMM-based clustering features for the data points, were generated based on the developed GMM: cluster label, assigned cluster likelihood score, and weighted average likelihood score.

Cluster label is represented by integers from 1 to k *(*k is the number of clusters selected in the GMM*)*. Cluster likelihood scores derived from the model measure how *irregular* each day (represented by a data point) is by calculating its deviation from the Gaussian mixtures. If we consider the center of each Gaussian as a typical routine, the farther out a point is in this Gaussian space, the higher the chances that the point represents an anomalous day or behavior.

The likelihood of a data point in a multivariate Gaussian distribution can be computed by calculating the probability of observing a point farther than the given point. In other words, the cumulative distribution function is evaluated at a given data point, which can be obtained using Mahalanobis distance metric. Note that the squared Mahalanobis distance from a point to the center of a Gaussian distribution has been proven to follow a chi-squared distribution with *p* df, where *p* is the number of variables [36]. Therefore, the likelihood of a point in the Gaussian distribution is equivalent to the cumulative probability of observing a value larger than the given Mahalanobis distance in a chi-squared distribution with *p* df.

The assigned cluster likelihood score of the data point was obtained as the probability of each point to its assigned cluster. The weighted average likelihood score was computed as the weighted (with the cluster’s corresponding weights) sum of the probability of a given point belonging to each of the Gaussian classes. Intuitively, the assigned cluster likelihood score measures how similar a day is to its closest routine. The weighted average likelihood score measures how similar a day is to all routines. As the weighted average likelihood score accounts for cluster weights, a point that is closer to a more populous cluster will be considered less anomalous. A 2D illustration of the likelihood scores is provided in Figure S1 in Multimedia Appendix 1.

#### PAM With Dynamic Time Warping Clustering

##### Model Introduction

GMM measure the similarity between observations (data points) using point-wise alignment of different features in the observation. However, the dissimilarity between 2 observations could be overestimated owing to an outlier (eg, because of faulty sensor measurements) or when there is a small time-shift or speed difference between observations. For example, 2 daily templates with a similar pattern but a shift of 1 hour would be expected to represent similar behavioral representations, but these templates would likely be considered dissimilar from the GMM. To allow flexible similarity assessments, we used dynamic time warping (DTW) to determine the optimal alignment of indices of the 2 time series that minimizes the distance between the time series [37]. The DTW distance can be paired with a distance-based clustering method such as a PAM clustering model [38]. The PAM model searches for k representative objects (medoids) from the data and creates clusters so that the total dissimilarity of points within clusters is minimized. We compared the number of clusters k based on the sum of the squared DTW distance of every data point with its cluster medoid and the elbow method (see more details in Multimedia Appendix 1).

##### Model Output

From the fitted PAM model, similar to the procedure after GMM fit, we generated three output features characterizing each data point: cluster label, assigned cluster distance score, and weighted average distance score. Similar to the GMM-based likelihood score computation, the cluster distance scores evaluate how dissimilar each object is from a representative data point or from all representative data points. The assigned cluster distance score is the DTW distance of each data point (representing a daily template) to its cluster medoid. A lower value indicates that a day conforms better to its most similar routine. Weighted average distance score was obtained by summing the DTW distance to all medoids scaled by the corresponding cluster sizes. A lower value indicates that a day conforms better to all possible routines. DTW distance from the previous day’s daily template was also calculated as a potential relapse predictor.

### Analyzing Cluster Results

After obtaining the output variables from the cluster models, we evaluated whether there were significant changes in any of these cluster output variables closer to relapse events. To quantify this change, we first defined different key periods to focus before a relapse. Similar to a previous study, we defined NRx as x days near relapse period (before the relapse event) and pre-NRx as all days before relapses that were not in NRx (healthy period) [21]. We evaluated the cluster outputs for NR7, NR14, NR20, and NR30 periods to test different window sizes. Cliff *δ* was computed to estimate the size of the change in the likelihood scores (GMM output) and distance scores (PAM model output) between the NRx and pre-NRx periods for each patient separately [39]. Cliff *δ* was chosen because of the nonnormality and variance heterogeneity of our data, for which the Cliff *δ* is a suitable metric. It is calculated as follows:



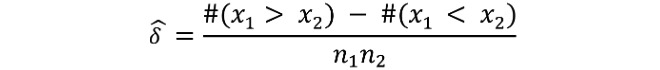



In the formula, #(x_1_ > x_2_) counts the number of values in group 1 (NRx period) that is larger than a value in group 2 (pre-NRx period) for all value pairs, and *n_1_* and *n_2_* are the sample sizes. This effect size ranges from −1 to 1, where 1 indicates that all values in the NRx period are larger than all values in the pre-NRx period, and −1 indicates vice-versa. As proposed by Romano et al [40], the effect can be considered to be nonnegligible if the absolute value is >0.147. Cliff *δ* is suitable to compare continuous variable output such as likelihood scores and distance scores.

### Relapse Prediction

#### Relapse Prediction Approach

**Figure 1:**
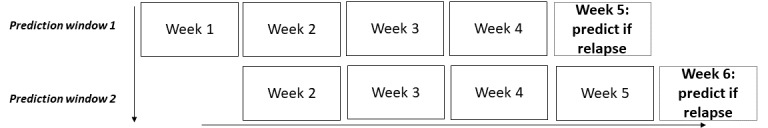
Sequential relapse prediction approach used in this study. Features are extracted from a period of 4 weeks to predict if a relapse might occur in the coming week.

We framed relapse prediction as a binary classification problem similar to the earlier studies [19,41]. On the basis of the mobile sensing features derived from a feature extraction window (current and immediately past observations from a patient), we predicted whether the patient is likely to experience a relapse in an oncoming period (prediction window). Similar to previous studies [19,41], we used a 4-week period as the feature extraction window and a 1-week period as the prediction window (Figure 1). Thus, mobile sensing observations from the past 4-week period were used in the relapse prediction model to predict whether there will be a relapse in the next week.

#### Features

##### Overview

Mobile sensing data contain features that characterize behavioral patterns in the relapse prediction model. In this study, we evaluated the contribution of the clustering features derived from the GMM and PAM models for the psychotic relapse prediction task. We briefly describe the baseline features (based on an earlier study [19]) and clustering-based features that are added to the relapse prediction model.

##### Baseline Features

These consist of all the features as used in the study by Lamichhane et al [19] along with distance-based and duration-based mobility features and screen use-based features. The CrossCheck data set contains information about when the screen of the patient’s smartphone is active. A single screen-use modality that represents the time spent using the phone (phone screen was active) was derived. Using this modality, the mean and SD of daily averages in a given feature extraction window were computed as features for the relapse prediction model. Similarly, for mobility-based features, we computed four different mobility modalities: distance traveled from home (home information obtained based on the clustering of the GPS locations), total movement, average time spent in a location, and total time spent at home. Then, for each mobility-based modality, we computed the mean and SD of the daily averages as features characterizing a feature extraction window.

##### Clustering-Based Features

We extended the baseline feature set with our proposed clustering-based features for the relapse prediction task. These features are listed in Table 1.

**Table 1:** Features used in relapse prediction models. Baseline features are derived from a previous study [19]. We evaluated if the clustering-based features could improve relapse prediction by complementing the daily behavioral rhythm change-based features represented in the baseline features.

Feature set and modalities		Features
**Baseline features**		
	Accelerometer magnitude; ambient light; distance traveled; call duration; sound level; and conversation duration	Mean daily template features (mean, SD, max, range, skewness, and kurtosis), SD template features (mean), absolute difference between mean and maximum template (max), distance between normalized mean templates, weighted distance between normalized mean templates, distance between normalized maximum template and mean template, and daily averages (mean and SD)
	10-item EMAs^a^	Mean and SD of EMA items in feature extraction window
	Screen use, distance-based mobility features: distance from home and total movement, and duration-based mobility features: time spent at a location and time spent at home	Mean and SD of daily averages in feature extraction window
**Clustering features**		
	GMM^b^ features	Mean and SD of GMM label and GMM likelihood scores, number of cluster transitions, and number of cluster states
	PAM^c^ features	Mean and SD of PAM label, PAM distance scores, and DTW^d^ difference from the previous day; number of cluster transitions; and number of cluster states
Demographic features		Age and education years

^a^EMA: ecological momentary assessment.

^b^GMM: Gaussian mixture model.

^c^PAM: partition around medoids.

^d^DTW: dynamic time warping.

#### Classifier

For our relapse prediction pipeline, we used a balanced random forest (BRF) classifier with a low overall model complexity (using 11 decision trees). As a classifier, BRF is suitable for learning from an imbalanced data set, as is the case in our relapse prediction task and provides meaningful prediction probabilities in different decision fusion schemes (eg, in situations where only a limited number of sensor modalities are available for a patient). The number of decision trees to be used was heuristically chosen to limit the model size (lower number of trees), while still having a number of trees to maintain diversity for the generalizability of the ensemble model. We used the BRF implemented in the imbalanced-learn library in Python [42], allowing the default unrestricted depth of trees and squared root of number of features considered for best split in the trees. Similar to the approach used by Lamichhane et al [19], features were quantized into discrete bins before being provided as input to the classifier. The number of bins was set as a hyperparameter, and for the set number of equal-width bins, the count of feature values in each bin was retained as the processed feature values. The approach of feature quantization was found to be helpful in relapse prediction, probably by ignoring small insignificant changes while retaining large feature variations representing significant behavioral deviations. We used leave-one-patient-out cross-validation for the evaluation of the model. The number of bins to be used was a hyperparameter for the classification model and was set with cross-validation within the training set (nested cross-validation). The number of bins for feature quantization considered in hyperparameter tuning were 2, 3, 4, 5, 10, and 15, and the tuning procedure is further described in Multimedia Appendix 1.

#### Relapse Labels

For our relapse prediction pipeline, as the relapse dates are not a fixed discrete event, a hard label and earlier indications of an oncoming relapse are also valuable, we considered the entire month preceding the date of indicated relapse as a relapse period for classification. Thus, any prediction of relapse within a 4-week period before the relapse was considered as a useful output from the prediction model, as has also been used in previous study on relapse prediction [21]. A relapse prediction generated up to a month before the relapse would be observable and potentially actionable for interventions, as behavioral changes associated with relapse could manifest up to a month preceding a relapse [18].

#### Personalization

**Figure 2:**
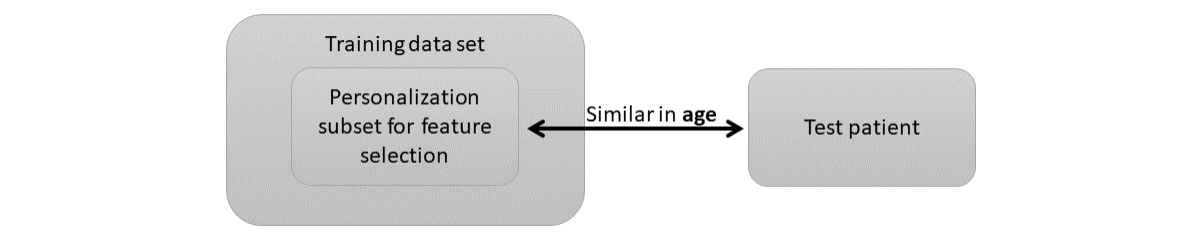
Personalization approach for the relapse prediction model [19]. A personalization subset, consisting of data from patients who are closest in age to the test patients, is used to identify the best feature sets, using which a (personalized) relapse prediction model can be trained.

Human behavior and behavioral change manifestations of relapse can be person-dependent. Lamichhane et al [19] proposed a method for personalizing a relapse prediction model based on feature selection adapted to a particular test patient. The adaptation occurs using a personalization subset. This is illustrated in Figure 2. For a test patient, within the leave-one-patient-out cross-validation approach, the data from patients closest in age to the given test patient comprised the personalization subset. We included age-based personalization as a first step toward personalized relapse prediction, as behavioral tendencies could be dependent on age, among other factors. Age has been reported to be a significant factor in univariate regression modeling of relapse behaviors in patients with schizophrenia [43], and age dependence of psychosocial functions, substance use behaviors, psychotic symptoms, hospitalization risks, and so on, have been reported in the context of psychotic relapse in patients with schizophrenia [44]. We evaluated the gains from age-based personalization compared with a nonpersonalized model to empirically establish if age-based personalization could be helpful in behavioral modeling and relapse prediction. As relapse incidents are rare, all the relapse incidents in the training data set were included as a part of the personalization subset. For training a classifier toward the test patient, the optimal features were selected using the personalization subset. We used this approach for training our relapse prediction model, and used the correlation between features and target labels as the feature selection criteria. The number of features to be selected was set as a hyperparameter in our classifier, and this dictated the threshold for correlation value used for feature selection. For example, if the number of features to be selected is 5, the threshold for correlation coefficient (absolute value) is selected such that top-5 features with the highest correlation with the labels are retained. The number of features to be used was selected from 3, 5, 10, and 15, and the features and size of the personalization subset was selected from 50, 75, 100, 125, 150, 200, and 300 for the hyperparameter tuning (further described in Multimedia Appendix 1).

#### Evaluation Metric

We evaluated relapse prediction performance to assess the contributions from clustering-based features. Any improvement in the relapse prediction performance when clustering-based features are added to the baseline features would establish the value of clustering-based features to represent behavioral trends and detect anomalies relevant for relapse prediction. Similar to the study by Lamichhane et al [19], we used F2 score for model evaluation to slightly prioritize recall over precision (detecting a relapse is slightly prioritized over generating a false positive). F2 score is given as follows:



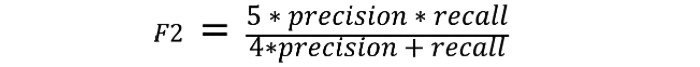



We also report the obtained precision and recall scores together with the F2 scores.

## Results

### Clustering Results

We trained GMM and PAM models to obtain cluster centers and identify different behavioral routine representations. The model selection procedure is explained in Multimedia Appendix 1, and model comparison metrics for GMM and PAM are plotted in Figures S2 and S3 in Multimedia Appendix 1. For GMM, after evaluating AIC and BIC scores, model selection was narrowed to 8 to 14 clusters with full covariance matrix. Among the models with equally good AIC and BIC scores, the models with 9 and 13 clusters achieved the best model stability and least overlap between Gaussian classes. The final model selection was 9 clusters because lower number of clusters allows for higher interpretability. The number of clusters for the PAM model was also selected to be 9 based on the distance dissimilarity metric and elbow method.

See Figures S4 and S5 in Multimedia Appendix 1 for the output from the GMM and PAM models, including cluster size, average likelihood scores (for GMM), distance scores (for PAM; Figure S4 in Multimedia Appendix 1), and kernel density plots that illustrate the distributions of likelihood scores and distance scores (Figure S5 in Multimedia Appendix 1).

**Figure 3:**
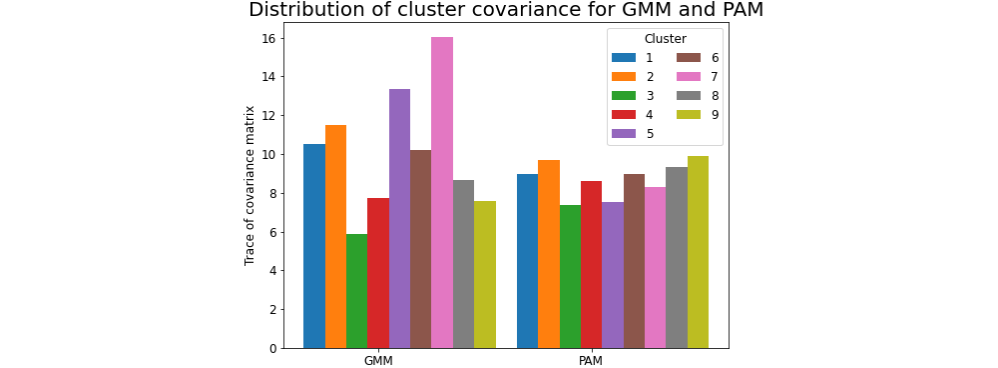
Trace of the sample covariance matrix for each cluster obtained with Gaussian mixture model (GMM) and partition around medoids (PAM) clustering approach. A low covariance matrix trace indicates more homogeneous clusters, that is, clusters with lower within-cluster variability.

To evaluate how well the days in each cluster conform to a routine—the one represented by the cluster center—we measured the spread of each cluster using the trace of the covariance matrix of all cluster samples. Results are illustrated in Figure 3. Clusters with small covariance trace had low within-cluster variability. The GMM cluster model resulted in a more extreme distribution of cluster spread (higher range of covariance trace) because it allowed the clusters to overlap (despite our model selection approach to limit overlaps), whereas the PAM model created partitions in the data.

**Figure 4:**
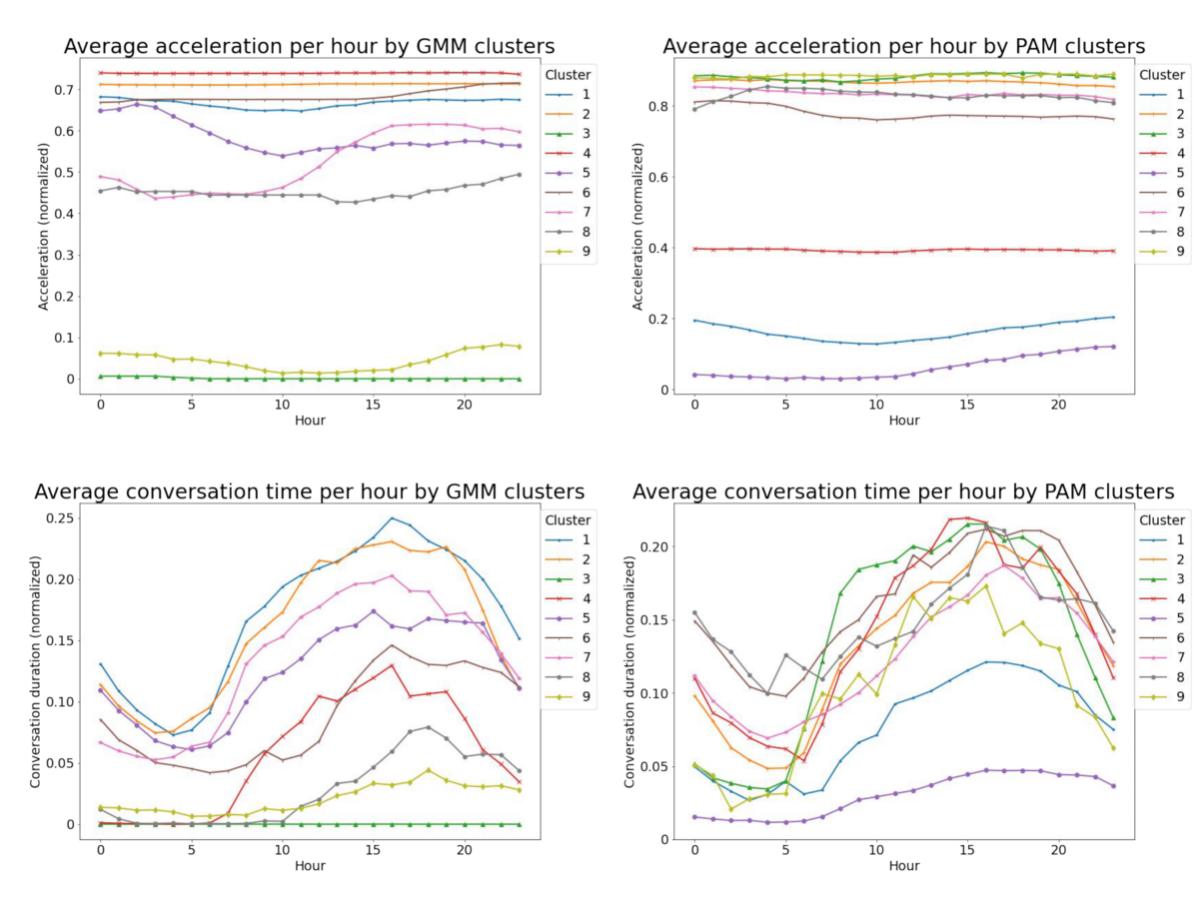
Average daily templates of two signal modalities acceleration (top) and conversation time (bottom) in the clusters obtained from the Gaussian mixture model (GMM) and partition around medoids (PAM) models. Different clusters capture different behavioral patterns.

By averaging all daily templates (data points) in every cluster, it was possible to observe the cluster profiles. For example, Figure 4 illustrates the average daily templates of two example signal modalities: acceleration and conversation. The GMM performed better in stratifying daily templates based on the overall level of activity in these signal modalities. The PAM model had high variance in each cluster because it allows for a more lenient dissimilarity measurement. Although the daily templates in each cluster have different levels of signal activity, they generally follow the same pattern as a normal circadian rhythm; for example, the conversation signal activity peaking during the day and being at minimum during the night.

Table 2 summarizes the average profile for each cluster, ordered from the most common to the least common cluster.

**Table 2:** All cluster profiles obtained from the GMM^a^ and PAM^b^ models in descending cluster size. Different clusters are associated with peculiar behaviors specific to that cluster as it can be observed from the typical profile of signal modality in that cluster.

Cluster size rank	GMM cluster profile	GMM cluster size (days)	PAM cluster profile	PAM cluster size (days)
1	No app use, high conversation and SMS text messaging, and other attributes are approximately average	5217	Low acceleration, conversation, volume, and sleep duration and very low variability in sleep and volume templates	3318
2	Highest app use and phone calls; high acceleration, conversation, SMS text messaging, distance moved, and volume; early wake up (at approximately 7 AM); and no sleep during the day	3993	High volume and SMS text messaging and constantly low sleep template	3300
3	Almost all sensor readings near 0	2580	Conversation and volume sharply increase after 6 AM, highest volume, low phone use before 7 AM, wake up at approximately 7 AM, and sleep at approximately 9 PM	2728
4	Highest acceleration, low phone calls, early wake up (at approximately 7 AM), and no sleep during the day	1883	High app use, SMS text messaging, and distance moved around midnight and below average acceleration	2699
5	High acceleration after midnight, high phone calls and SMS text messaging, high overall volume even at night, and late sleep and wake up	1484	Lowest acceleration (close to 0) and app use and constantly high screen time and sleep duration	2378
6	Below average volume and distance, wake up after 11 AM, and sleep during the day	1298	High phone calls and SMS text messaging, screen time sharply increases after 6 AM, wake up at approximately 9 AM, sleep at approximately 11 PM, and awake during the day	1686
7	Activity level and phone use are high during the day, inactive at night, short sleep duration, high number of phone calls, and acceleration increases after 3 PM	1046	Below average screen time and long sleep time (wake up around noon)	1405
8	No app use; low conversation, SMS text messaging, and volume; and long sleep even during the day	523	Low phone call, SMS text messaging, and screen time; high volume at night; and constant long sleep (wake up in the afternoon)	752
9	Accelerometer readings close to 0; low app use, conversation, and volume; phone screen is constantly on; and long sleep duration even during the day	412	High app use and distance moved during noon; templates in this cluster have high dissimilarities	170

^a^GMM: Gaussian mixture model.

^b^PAM: partition around medoids.

### Association With Relapses

**Figure 5:**
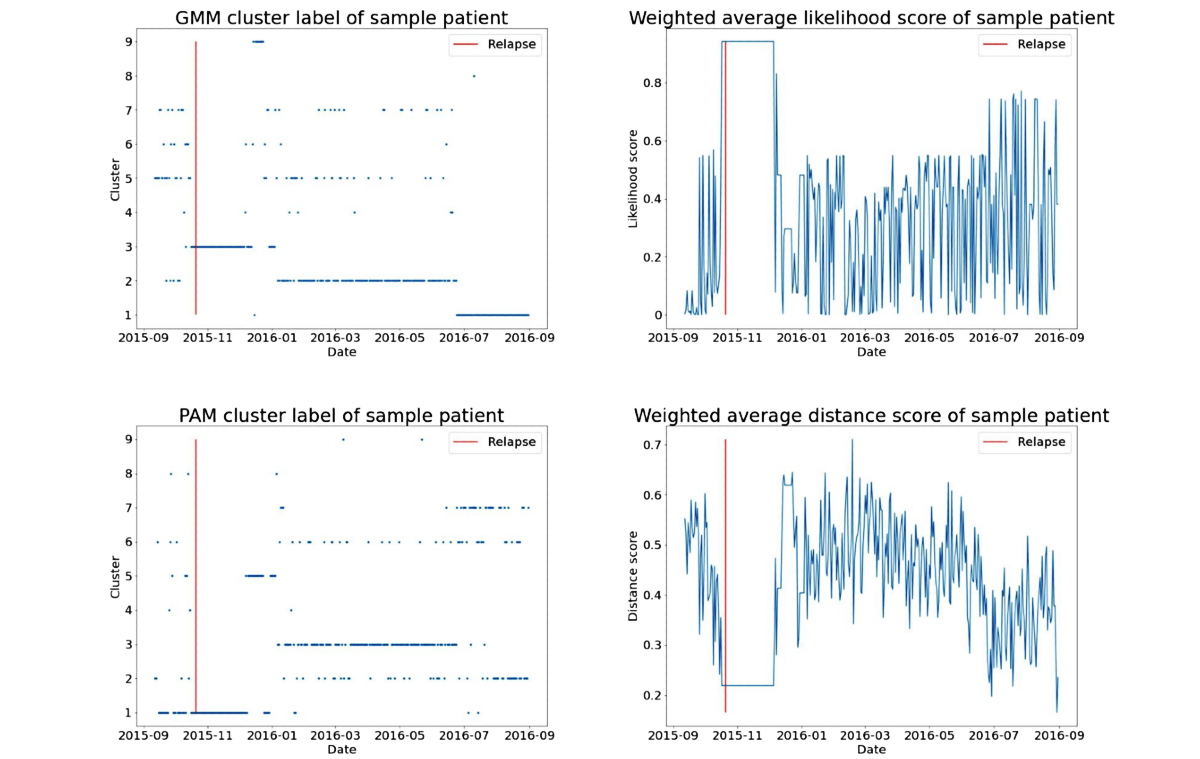
Time series plots of cluster assignment as obtained from the Gaussian mixture model (GMM) and partition around medoids (PAM) models (left pane) and weighted average likelihood score and distance score of a sample patient (right pane). Changes in cluster features are seen near the relapse instance (shown with the vertical red line).

Of the 27 relapse events in total, clustering features were missing before 3 (11%) events owing to missing signal modalities. For 11 (46%) of the remaining 24 relapses, anomalies in clustering features were observed qualitatively in the time series of these features before and after the relapse. Most of these anomalies represented a transition to a cluster with inactive sensor readings; for example, GMM cluster 3 and PAM cluster 1 (Figure 5). We hypothesized that the patients, for whom we see their assigned cluster labels near relapse period being assigned to the cluster of inactive sensor recordings, most likely had their phone turned off a few days before the relapse. This transition to an inactive cluster was associated with an increase in likelihood scores (GMM-based feature) and a decrease in distance scores (PAM model-based feature) because these clusters were more compact and points did not deviate very much from the cluster centers.

**Figure 6:**
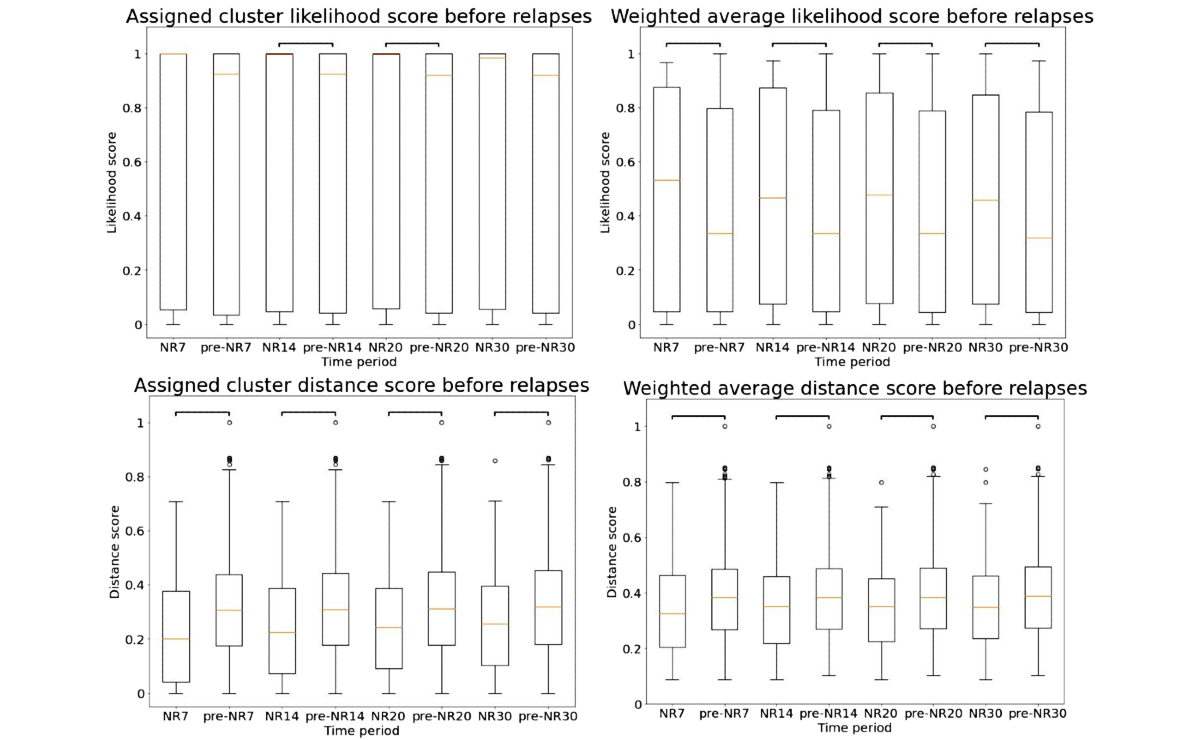
Boxplot of the clustering features (likelihood scores from Gaussian mixture model on top and distance scores from partition around medoids model at the bottom) in x days near relapse (NRx) and all days before relapses not in NRx (pre-NRx) periods. Bars indicate nonnegligible effect size.

Our cluster analysis between the NRx and pre-NRx periods showed that, on average, likelihood scores increased and distance scores decreased closer to relapses (Figure 6). This trend was robust with respect to different window sizes, and the largest change was observed with NR20 window size. Asterisks indicate that the absolute Cliff *δ* value between the 2 periods is >0.147 (ie, effect is nonnegligible; refer to *Analyzing Cluster Results* in the *Methods* section). Note that the plots were made using all patients’ data collectively. Individual patient’s plots would show a large difference between the near-relapse window and healthy period. Average Cliff *δ* values across all relapse events are presented in Table S1 in Multimedia Appendix 1.

We evaluated the relapse prediction pipeline discussed in *Relapse Prediction* in the *Methods* section, with and without the clustering-based features. The highest F2 score of 0.23 was obtained when the baseline features were complemented with the clustering-based features, which was significantly higher than the F2 score from random classification baseline (0.042) and the F2 score obtained using the baseline features only (0.18).

### Significant Features

With the best relapse prediction obtained using all features, we identified the most important features within this feature set based on how often a feature was selected in the leave-one-patient-out cross-validation. The selection count for a feature was incremented by 1 if it was selected for use in a particular cross-validation loop for a test patient. It should be noted that the number of features selected in each cross-validation loop was different because the number of features was a hyperparameter selected using nested cross-validation. Then, we normalized the total selection count of each feature at the end of the cross-validation by the number of cross-validation loops. The results obtained are presented in Table 3.

**Table 3:** The top 10 significant features in the relapse prediction pipeline based on the entire feature set (baseline and clustering-based features). The frequency of selection of a particular feature across the cross-validation loop is used to assess the most significant features for relapse prediction. It is to be noted that different numbers of features are selected in each cross-validation loop, as the number of features to be used is a hyperparameter tuned with a nested cross-validation loop.

Features	Frequency (normalized)
Baseline feature–distance template skewness	0.19
Clustering feature–mean PAM^a^ label	0.17
Clustering feature–mean PAM weighted distance	0.14
Baseline feature–conversation template skewness	0.14
Clustering feature–number of transitions	0.12
Clustering feature–SD GMM^b^ label	0.10
Clustering feature–SD PAM label	0.10
Clustering feature–mean GMM assigned cluster likelihood	0.10
Baseline feature–conversation template kurtosis	0.08
Baseline feature–volume template range	0.08

^a^PAM: partition around medoids.

^b^GMM: Gaussian mixture model.

## Discussion

### Principal Findings

In this study, we used clustering models to obtain behavioral representation from mobile sensing data, which could be useful for relapse prediction. The two clustering models explored in this study, GMM and PAM, grouped observations using different notions of distance or similarity between data points, and therefore, captured different behavioral representations (Table 2; Figure 4). These representations can be useful in downstream applications such as relapse prediction.

The GMM defines distance based on one-to-one matching between the hourly observation of mobile sensing data in the daily template. The clusters identified from the GMM have a widely varied distribution of cluster spread (Figure 3). With some compact clusters (represented by low cluster covariance) being identified within the GMM, the remaining data points that do not belong to any of these compact clusters are considered as a large-spread cluster with no typical cluster profile. These large-spread clusters also contain the compact clusters (cluster overlaps); a point belonging to compact clusters also shows high likelihood of belonging to the large-spread cluster. As we wanted the clusters to capture distinct behavioral trends, we evaluated Bhattacharyya distances to identify the best clustering model with least overlap between identified clusters. The PAM model with DTW distance allows a more lenient match of daily templates of behaviors as represented by the mobile sensing-based features. Such a lenient matching fits the context of this study because DTW is able to adjust for spikes, speed differences, or time shifts when evaluating dissimilarity between 2 daily templates of behaviors. However, the clusters obtained from the PAM model contain more dissimilarities. Then, it is more difficult to summarize the cluster profiles for qualitative model interpretation.

Overall, GMM-based modeling can identify highly dense or populous clusters with very specific behaviors associated with these clusters and some dispersed clusters that do not have a typical cluster profile. For example, cluster 3 and cluster 9 identified from the GMM (Table 2) represented 2 types of typical routines. Cluster 3 from the GMM had almost all sensor readings, except sleep, close to 0, likely representing an inactive or sedentary day, and cluster 9 had the days with the phone screen always turned on, likely representing a day with high mobile phone use. The PAM model also had a cluster with mostly inactive days and constantly long screen time (cluster 5). However, this cluster had higher cluster variance. When the average cluster profile of this cluster was observed (Figure 4), some days that did not strictly follow the patterns of inactive day and long screen time were also assigned to the cluster. In terms of behavioral features, this implies that clusters obtained from a PAM model are likely to cluster together the behaviors that do not always show homogeneity based on qualitative observations. This is because of the flexibility of the PAM model in allowing unparalleled alignment between behavior time series. Nonetheless, it might be beneficial to consider PAM-based modeling for the previously mentioned features: ability to discount spikes and speed differences or time shifts when evaluating dissimilarity between 2 daily templates of behaviors. Similarity (or dissimilarity) between behaviors may not always be fully represented by hourly alignment and comparison of mobile sensing data across days.

The behavior of a particular day, represented by the mobile sensing data template for that day, was characterized in a clustering model with different clustering-based features such as Gaussian likelihood and DTW distance to the cluster centers. Days with assigned cluster likelihood scores close to 1 and assigned cluster distance scores close to 0 tend to belong to a dense cluster with a small spread. For example, cluster 3 from the GMM and cluster 5 from the PAM model have the highest likelihood and lowest distance to its assigned cluster, respectively (Figure S4 in Multimedia Appendix 1). They also have low within-cluster variability, as measured by the trace of sample covariance (Figure 3). On the other hand, days characterized by low likelihood scores and high distance scores tend to be more dispersed and do not conform well to a specific routine. For example, cluster 5 and cluster 7 in GMM and cluster 9 in the PAM model have such properties. Overall, GMM clustering and PAM clustering tend to produce clusters with different behavioral representations in the assigned clusters, and this is reflected in the clustering-based features such as likelihood scores and cluster distance that are assigned to characterize each day.

In terms of relapse prediction, clustering-based features can capture long-term behavioral trends across the patients. This representation can complement existing approaches to behavioral representation for psychotic relapse prediction in schizophrenia; for example, based on the use of daily behavioral rhythm change features as proposed by Lamichhane et al [19]. We compared the clustering-based features before and near the relapse periods and observed significant differences in some features. This was also seen qualitatively in a time series plot of clustering-based features, indicating that an upcoming relapse for a patient is associated with changes seen in clustering-based features (Figure 5). Clustering-based features were helpful in relapse prediction models (Table 4).

When clustering-based features were used together with daily behavioral rhythm change features, a significant gain in relapse prediction performance was obtained (F2 score improved from 0.18 to 0.23). These F2 scores and the associated improvements are significant, considering that a random classification baseline gives an F2 score of 0.042 on average. A Wilcoxon signed-rank test on performances in multiple classifier initializations for classification with and without clustering features yielded a significantly high score for classification when clustering features were included (*P*=.002). Clustering-based features were among the top features when significance of features for the relapse prediction task was evaluated (Table 3). Features such as mean cluster labels and number of transitions of labels were among the top (most frequently selected) features. Thus, both the information about which behavioral clusters the observations from the current period of monitoring belong to (likely representing behavioral clusters that are not normal behaviors) and how often transitions between different behavioral clusters occur (representing the patient showing frequent behavioral variations) are likely predictive of an oncoming relapse. Clustering-based features alone also proved to be valuable for relapse prediction. GMM-based and PAM-based clustering features only used in the relapse prediction pipeline led to an F2 score of 0.16 and 0.16 for relapse prediction, respectively (Table 4). Therefore, clustering-based features are found to be a useful approach to obtain behavioral representations and can be used in clinical applications such as relapse prediction.

**Table 4:** Relapse prediction performance with different feature sets. The baseline features introduced in the previous study [19] are complemented with clustering-based features for evaluation. The performance of both the GMM^a^-based and PAM^b^-based feature sets are also separately evaluatedc.

Method	F2 score (precision/recall)
All features	0.23 (0.063/0.662)
Baseline features [19]	0.18 (0.055/0.400)
Clustering features	0.14 (0.035/0.487)
GMM features	0.16 (0.042/0.487)
PAM features	0.19 (0.042/0.525)
GMM+baseline features	0.19 (0.052/0.525)
PAM+baseline features	0.16 (0.045/0.438)

^a^GMM: Gaussian mixture model.

^b^PAM: partition around medoids.

^c^Random classification baseline: mean score 0.042 (SD 0.020).

### Comparison With Previous Studies, Limitations, and Future Research

To the best of our knowledge, this is the first study that used clustering analysis to group behavioral patterns of individuals with schizophrenia. Compared with previous studies that used hourly data to train the relapse prediction models, our study, based on clustering features to represent different behavioral patterns, has better model interpretability. Clustering analysis allows clinicians to understand different types of patient routines and their frequencies. In terms of schizophrenia, cluster transitions observed before relapses could suggest which types of behavior are potential relapse-related behavioral signatures. Then, intervention strategies to prevent relapses can be made accordingly.

Researchers have studied how missing data are related to relapses and anomalies in mental health conditions. In the data set that we used for evaluation in this study, some passive-sensing daily templates have consecutive hours with missing data from almost all signal modalities. In an anomaly detection study, Adler et al [21] used mean imputation, whereas here, we filled missing values with zeros. Filling missing data with mean values may ignore the potential relationship between missing data and anomalies. In reality, it is highly possible that outpatients may turn off their phone when they experience relapse symptoms. We observed that there are more days from an inactive sensor reading cluster closer to relapses. The increased prevalence of inactive days also caused likelihood scores to increase and distance scores to decrease before relapses. Initially, we hypothesized that adhering to any routine or cluster center might reduce the risk of relapse, but it turned out that some routines, such as missing sensing data, are actually associated with a higher risk of relapse.

Although the clustering features successfully improved relapse prediction results, the only observable relapse signature was an increase in likelihood score or a decrease in distance score and a transition to an inactive cluster. For the relapse events that were not indicated by sensor inactiveness, we did not find any nontrivial changes in any specific feature before the relapse. Similarly, the relapse prediction performance with the best F2 score of 0.23 is relatively low. However, investigations of mobile sensing-based relapse prediction in mental health disorders are relatively new, and further improvements in this field can be expected as more data sets become available and improvements in machine learning models for the specific task of relapse prediction are made. In the study by Borges et al [45], relapse prediction in bipolar disorder was developed using clinical assessment features during patient visits. A high F score (F1) of up to 0.80 was reported. The relapse rate was quite high (relapse in >60% of the included patients) in the data set used by the authors, and the relapse prediction was performed at a patient level (instead of a weekly prediction model in free-living conditions, as in our case), which might have led to high performance.

In this study, we obtained patient-independent clusters; that is, generalized behavioral clusters, by pooling data from all the patients. We generalized that there are certain types of routines across all outpatients with schizophrenia. Future studies can focus on establishing personalized cluster models. As suggested in the study by He-Yueya et al [25], every patient’s relapse signatures and the extent to which they adhere to their daily routine are different. The same study found that individual-level models can achieve better performance in predicting symptom severity. Our model also found that participants have different routines, as their frequencies of staying in different clusters largely varies. Moreover, although most patients had high likelihood scores and low distance scores closer to relapses, some other patients demonstrated the opposite trend. Generalized behavioral models might not fully represent and discount the effect of different confounding variables such as job type, sex, current health, and so on, which could impact behavioral trends. Although we used model personalization in relapse prediction, we considered only age as a covariate of behavioral trends. Personalized cluster models that account for different aspects of interpersonal differences would further help mitigate possible biases in behavioral representations owing to confounding variables. Personalized relapse prediction models will also be required to test the effectiveness of the individual-level clusters. However, sufficient data for each new patient are needed to find cluster models specific to the patient, and thus, clinical deployment for new patients will be delayed. Cluster adaptation from generalized cluster models to personalized cluster models, as more patient-specific data become available, needs to be investigated in future studies.

### Conclusions

In this study, we proposed a methodology to compute clustering models on 24-hour daily behavior of outpatients with schizophrenia and showed that information extracted from the cluster model improved relapse prediction. New features were generated from the cluster models by measuring the deviation of every observation from the cluster centers representing typical behavioral patterns. Two different clustering models were investigated. The GMM allows for cluster overlap and has a more extreme cluster dispersion. The PAM model with DTW distance creates partitional clusters that are more generalized toward new data, but fails to identify dense clusters. The clustering-based features helped to improve relapse prediction model performance. In future studies, we will further investigate personalized clusters and relapse prediction models.

## Appendix

Multimedia Appendix 1 Additional information, figures, and tables of clustering model selection, relapse prediction model selection, clustering, and relapse prediction results.

## Abbreviations

AIC: Akaike information criterion
BIC: Bayesian information criterion
BRF: balanced random forest
DTW: dynamic time warping
GMM: Gaussian mixture model
NRx: x days near relapse period
PAM: partition around medoids
