# Supplementary material for: Predicting Psychotic Relapse in Schizophrenia With Mobile Sensor Data: Routine Cluster Analysis
Source: JMIR Mhealth Uhealth. 2022 Apr 11;10(4):e31006. doi: 10.2196/31006 (PMC9039818; doi:10.2196/31006)
Supplement: Multimedia Appendix 1 [file mhealth_v10i4e31006_app1.pdf]

GMM Model Illustration

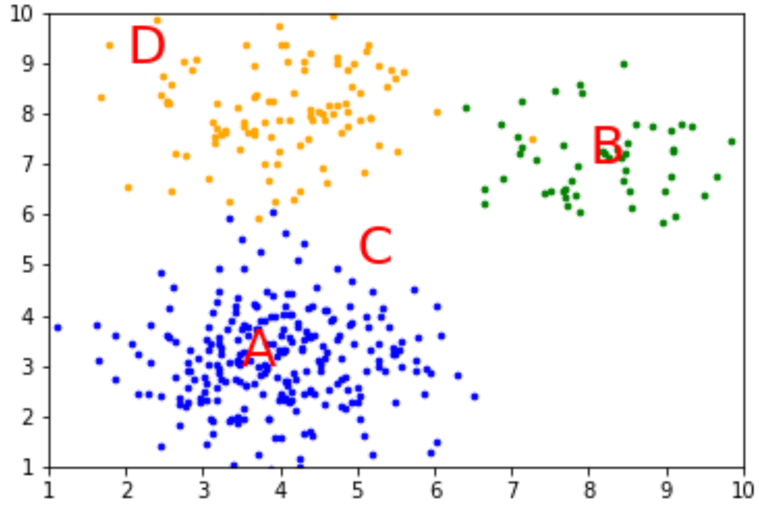

Figure S1: 2-D illustration of Gaussian mixture and likelihood scores (cluster likelihood and weighted average likelihood) computed for different example points. Point A and Point B have similar cluster likelihood scores as they lie near their respective cluster centers but Point A has higher weighted average likelihood as it belongs to a larger cluster. Point C and Point D have similarly lower cluster likelihood scores (lying farther from their closest cluster centers) but Point D has the lowest average likelihood score as it is farthest away from all the cluster centers. An anomalous behavior (a day with atypical behavior) will likely resemble Point D.

GMM Model Selection

Two parameters needed to be selected for the GMM model: the number of clusters *k* (ranging from 5 to 20) and the covariance matrix type (spherical, tied, diagonal, full). First we computed Akaike information criterion (AIC) and Bayesian information criterion (BIC) scores of all the candidate models (obtained using all the combinations of the possible covariance matrix types and the number of clusters). A main issue exists when training a GMM model, as also observed in our evaluation. Large cluster overlaps between gaussian components with low and high variance could be obtained, leading to non-informative clusters as it fails to identify a specific routine. To address this problem, for each model selection, we computed the average pairwise Bhattacharyya distances [44] between all fitted Gaussian distributions to evaluate the difference between the generated clusters. Larger value indicates larger separation and less overlap between clusters. The k-means++ [45] approach was utilized to determine the model initialization condition. This approach randomly selected the first centroid, computed clusters, and assigned the farthest point in the cluster model to be the next centroid, until *k* centroids were found, which were used as the initial centroids of the GMM model. To evaluate model stability, each model selection was trained five times with different randomizations. The mean

and standard error of each metric was illustrated in Figure S2. The optimal GMM model selected has high stability, low overlap, low AIC, and BIC.

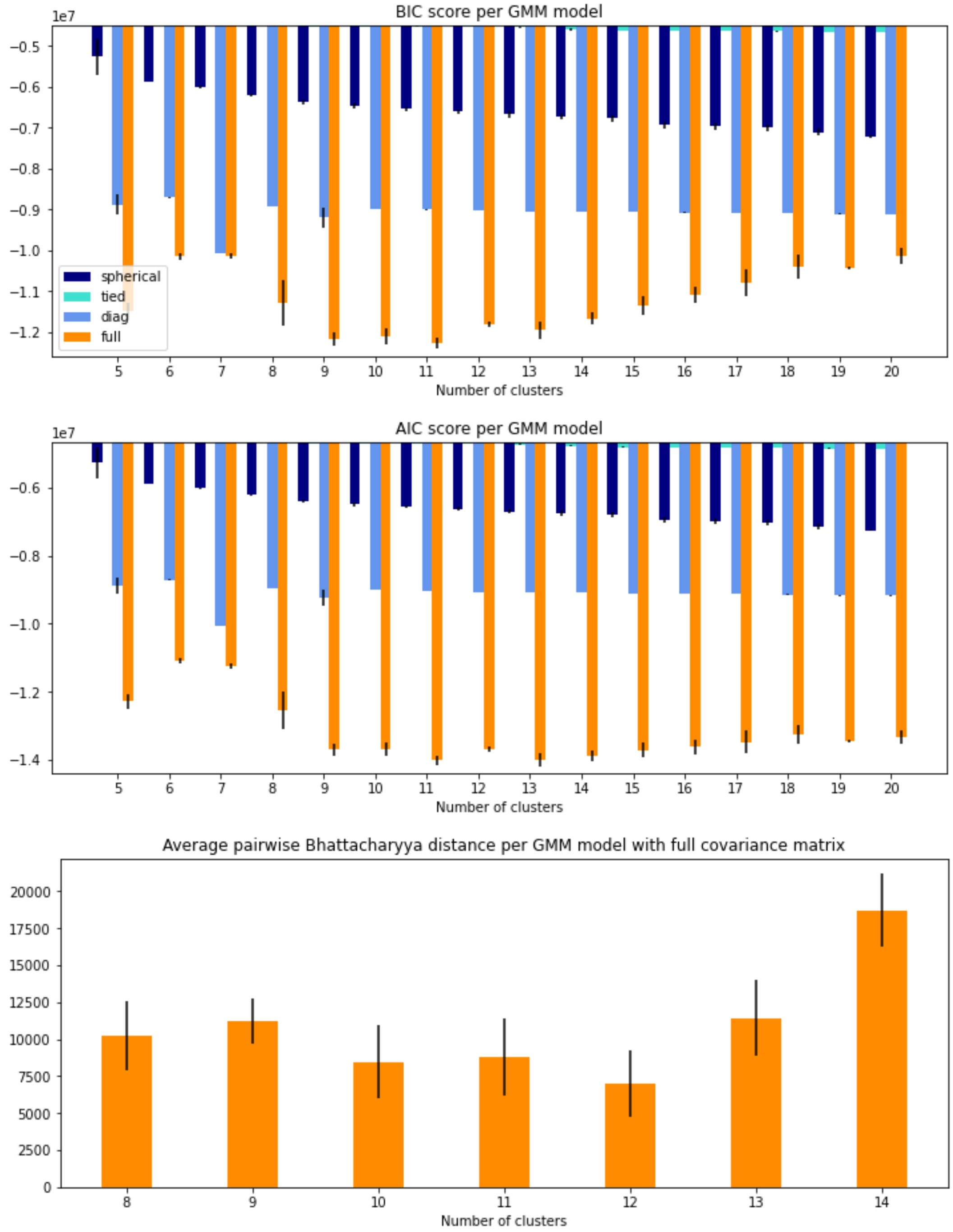

Figure S2: BIC scores, AIC scores, and average pairwise Bhattacharyya distances for each GMM model selection.

PAM Model Selection

We considered the number of clusters *k* ranging from 5 to 20, in the same range as those considered within the GMM model. For each possible number of clusters, we trained the model

five times with different initializations, and computed the sum of the squared DTW distance of every data point to its cluster medoid. The average sum of squared distance and the standard error were plotted in Figure S3. The final model was selected using the elbow method, which means the cluster number is optimal when adding one more cluster does not contribute too much to reducing the sum of squared distance.

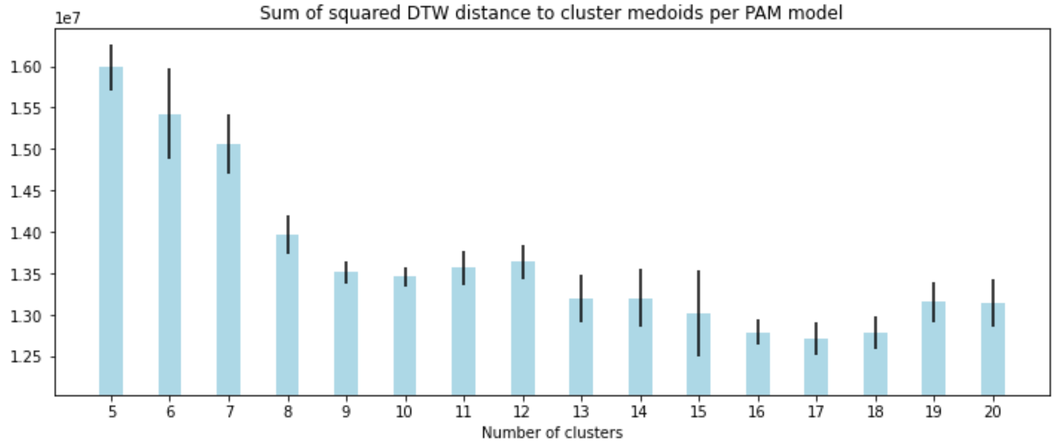

Figure S3: sum of squared DTW distance for each PAM model selection

Relapse Prediction: Hyperparameters for the classifier

Our relapse prediction pipeline using the Balanced Random Forest (BRF) as classifier has a few hyper-parameters namely: the number of bins to quantize features, size of personalization subset, and number of features to be selected for model training. We set the value of these hyper-parameters based on the cross-validation results obtained with different sets of parameter values within the training set. A nested cross-validation with random k-fold (k=10) within the training set was used to identify the hyper-parameter to be used for a given patient in the test set. We used a random k-fold cross-validation in the inner loop as leave-one-patient-out cross-validation would be computationally costly (number of folds would be 61 in the inner loop also). A random k-fold partition in the inner loop could still be helpful to identify good hyperparameters when a higher number of folds are used since most of the patients would appear in the test set in one of the folds and the discovered hyperparameters are thus generalizable. The parameter values considered for identifying the best parameters were: [2, 3, 4, 5, 10, 15] for the number of bins, [50, 75, 100, 125, 150, 200, 300] for the size of personalization subset, and [3, 5, 10, 15] for the number of features to be selected.

Clustering and Relapse Prediction Results

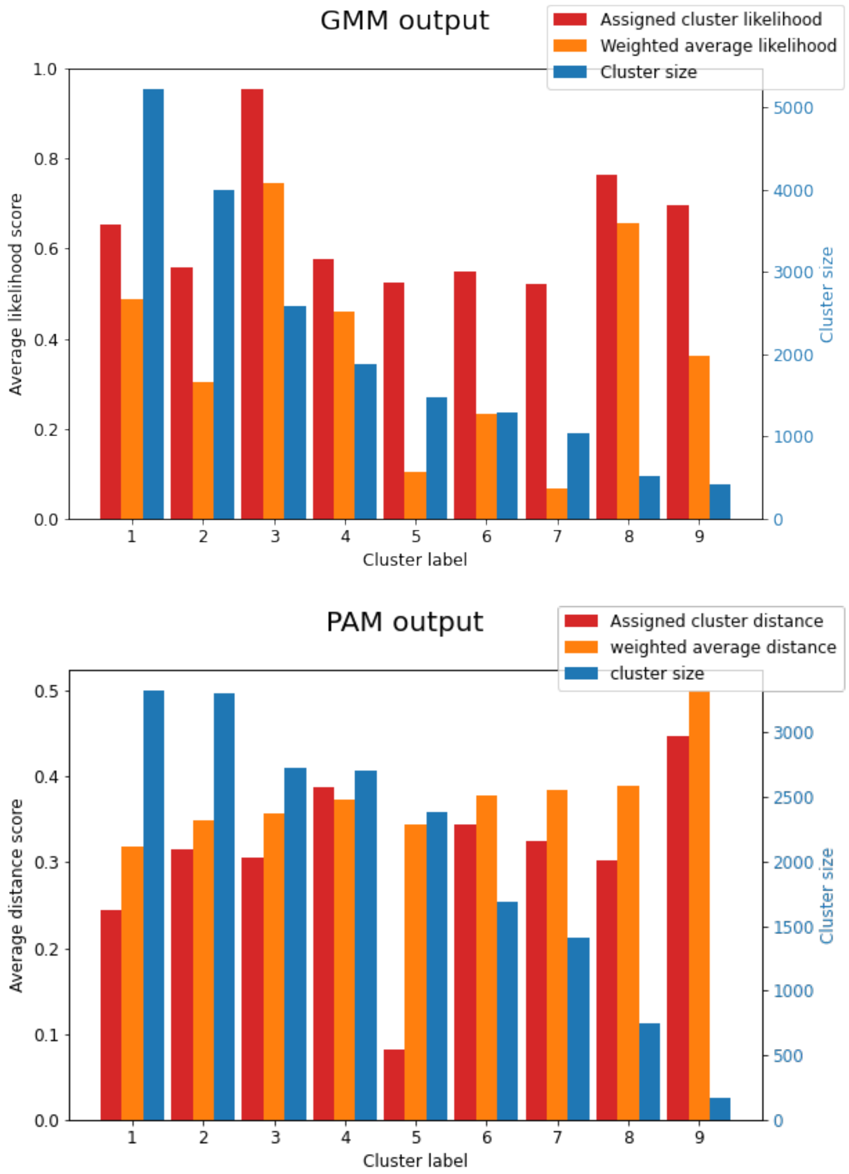

Figure S4.A (top): average assigned cluster likelihood score, average weighted average likelihood score, and cluster size of each GMM model cluster. Figure S4.B (bottom): average assigned cluster distance score, average weighted average distance score, and cluster size of each PAM model cluster.

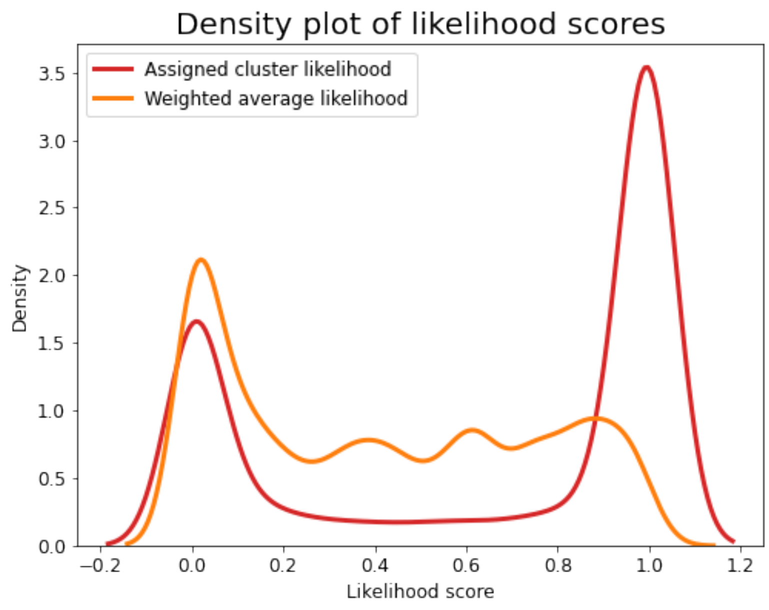

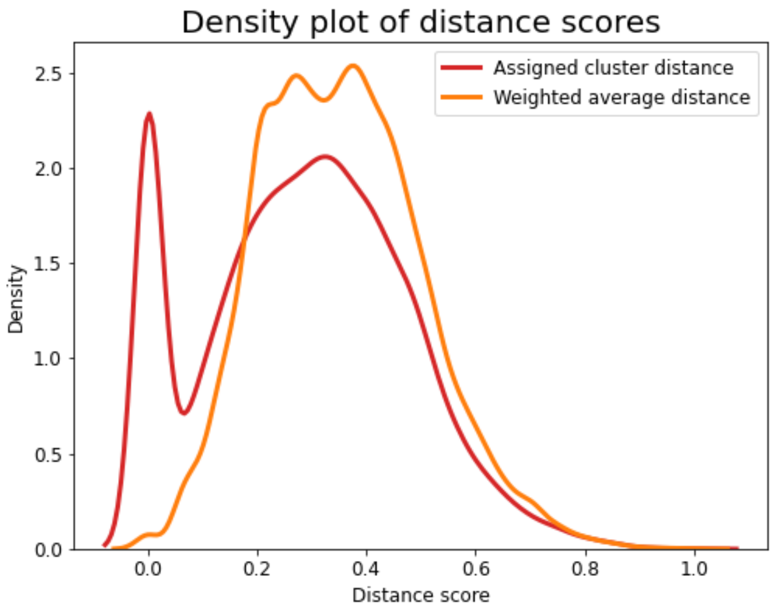

Figure S5.A (top): kernel density plot of assigned cluster likelihood score and weighted average likelihood score from the GMM model for all sample data. Figure S5.B (bottom): kernel density plot of assigned cluster distance score and weighted average distance score from the PAM model for all sample data.

	NR7 vs. pre-NR7	NR14 vs. pre-NR14	NR20 vs. pre-NR20	NR30 vs. pre-NR30
Assigned cluster likelihood (cliff's delta)	0.1346	0.1681	0.1963	0.1129

Weighted average likelihood (cliff's delta)	0.2023	0.2416	0.2552	0.2473
Assigned cluster distance (cliff's delta)	-0.1598	-0.2150	-0.2504	-0.2386
Weighted average distance (cliff's delta)	-0.1787	-0.1862	-0.2098	-0.2171

Table S1: differences in clustering features between the NRx (x days near relapse) and the pre-NRx (all days before relapses not in NRx) periods.

	**F2 score**	
**Method**	**With personalization (age-based)**	**Without personalization**
All features	0.23	0.14
Baseline features	0.18	0.14
Clustering features	0.14	0.11
GMM features	0.16	0.13
PAM features	0.16	0.16
GMM + Baseline features	0.19	0.13
PAM + Baseline features	0.16	0.14

Table S2: Effect of age-based personalization on relapse prediction performance. A higher F2 score in relapse prediction is obtained when age-based personalization is used compared to when no personalization is used.
